# Campaigning an educational website on ageism: lessons from a failed attempt

**DOI:** 10.1093/geront/gnaf264

**Published:** 2025-11-21

**Authors:** Liat Ayalon, Sarit Okun

**Affiliations:** Louis and Gabi Weisfeld School of Social Work, Bar Ilan University, Ramat Gan, Israel; Louis and Gabi Weisfeld School of Social Work, Bar Ilan University, Ramat Gan, Israel

**Keywords:** Social campaigns, Ageism, Older adults, Advertisement, Social media

## Abstract

**Background and Objectives:**

Ageism, defined as stereotypes, prejudices, and discrimination based on age, significantly impacts the health and well-being of older persons. It also has tremendous financial and social impacts on society at large. In response, this study evaluates a digital marketing campaign aimed at raising awareness of ageism through an educational website, “A site for all ages” (no2ageism.com).

**Research Design and Methods:**

The researchers implemented a mixed-methods approach: Study 1 utilized an online experiment to measure ad effectiveness (positive vs negative age messages) in attracting website traffic; Study 2 surveyed website visitors’ perceptions of the ads; and Study 3, which was added due to the low response rate to Study 2, relied on an online survey to assess perceptions of the ads. Data were analyzed using descriptive statistics and thematic analysis.

**Results:**

Following the introduction of paid ads, Study 1 showed an increase in website traffic, but not in engagement, with a moderate preference for the positive ad. Study 2 revealed that curiosity was the main drive behind clicking ads, while Study 3 demonstrated that older individuals, women, and those with higher educational level, preferred the positive ad highlighting inclusivity in their message.

**Discussion and Implications:**

This study resulted in successful traffic generation, although engagement with the website was limited, when ads were used. The limited engagement with the website, despite an increase in traffic, represents a failure given the tremendous efforts put into featuring an interactive, engaging website. Lessons learned can improve campaign design and implementation.

Ageism defined as stereotypes, prejudices, and discrimination toward people of all ages has a dramatic impact on the health and well-being of older persons and society at large (Ayalon & Tesch-Römer, 2018; World Health Organization, 2021). Given its detrimental public health effects, the World Health Organization (WHO) has embarked on a global campaign to combat ageism (WHO, 2021). In support of the global campaign, we developed an educational website on ageism to provide the public with the most up-to-date, evidence-based information on the topic (no2ageism.com). This study aims to enhance evidence in support of social campaigns to eradicate ageism. In this article, the researchers describe findings from an online marketing campaign which aimed to increase traffic to and engagement with the educational website.

## Ageism as a public health concern

Ageism is a multidimensional construct which manifests in a variety of contexts (Ayalon & Tesch-Römer, 2018; Iversen et al., 2009). At the individual level, ageism operates via the internalization of negative age stereotypes throughout the life course and their redirection toward oneself in older age (Ayalon & Tesch-Römer, 2017; Levy, 2009). At the interpersonal level, ageism is manifested in social interactions or their absence because of one’s chronological age (Nussbaum et al., 2005), whereas at the institutional level, ageism operates in laws and policies (Lloyd-Sherlock et al., 2016).

The prevalence of ageism is substantial. An analysis based on 57 countries has shown that one in two people reports ageist attitudes (Officer et al., 2020). Likewise, a study based on the European Social Survey has shown that one in three Europeans reports the experience of ageism (Ayalon, 2014). Ageism has shown to have a substantial impact on health and well-being and even serves as a higher risk for mortality (Chang et al., 2020). A recent review on the impact of ageism on health has shown that it exists in all 45 countries in which it was examined (Chang et al., 2020). The effects of ageism are not unique to the healthcare system though. It also operates in many other contexts including the workforce (Previtali et al., 2022), climate change (Ayalon & Roy, 2023), media (Aharoni Lir & Ayalon, 2022), art (Chen et al., 2022), and technology (Chu et al., 2022). For instance, in the workplace (Ayalon, Perel-Levin, et al., 2021), there is research to show that older persons are more likely to retire early when faced with discrimination. They also are less likely to be hired or promoted on the job. In the context of climate change, ageism might exacerbate conflict between the generations and hamper cooperation around climate change issues (Roy & Ayalon, 2022, 2024). Ageism is also highly present in the case of technology, with designers often failing to design interventions to accommodate older persons’ needs (Mannheim et al., 2023). This is further intensified by ageist messages and behaviors directed toward and internalized by older persons (Köttl et al., 2021). Ageism has a substantial impact on society at large, which can even be quantified financially. The annual cost of ageism is estimated at $63 billion in the U.S. healthcare system (Levy et al., 2020), whereas the cost of ageism in the workforce is estimated at $850 billion (Terrell, 2020).

### Interventions to eradicate ageism

In response to the significant public health impact of ageism, the WHO embarked on a global campaign to combat ageism. As part of the campaign, the WHO compiled available evidence on ageism and launched the first ever global report on ageism (WHO, 2021). The report identified four possible strategies to combat ageism. The first two are supported by research evidence as documented in a systematic review on the topic (Burnes et al., 2019). Specifically, educational interventions which provide people with information about older age and aging and intergenerational contact interventions, which bring together older and younger people around a common goal have shown to result in reduced ageist attitudes, improved knowledge, and comfort around older persons (Burnes et al., 2019). The report also determined that policy and legislations to reduce ageism represent an effective tool (WHO, 2021), although evidence concerning the impact of policy as a tool to combat ageism is still equivocal (Tesch-Roemer & Ayalon, 2023). The fourth strategy identified by the WHO as still lacking evidence in the fight against ageism is that of social campaigns (WHO, 2020).

### Social campaigns to eradicate ageism

Social campaigns are designed to change attitudes, feelings, and behaviors by raising awareness of social problems. Although the goal of campaigns might be behavioral change, most campaigns focus on raising awareness and knowledge. There are many examples of social campaigns ranging from attempts to change health behaviors (Ghahramani et al., 2022) to changing attitudes toward marginalized groups such as people with mental health conditions (Walsh & Foster, 2020). Many of these campaigns have relied on the provision of educational information to change attitudes (Kite et al., 2020; Tattan-Birch et al., 2020).

In the context of ageism, a recent review has shown that most campaigns were not supported by a research component and their effects on ageism were largely unknown (WHO, 2020). This is unfortunate given the many advantages inherited in social campaigns, which tend to reach a wide audience at a relatively low cost. The recent review of social campaigns to tackle ageism has identified multiple shortcomings that possibly prevent ageism campaigns from becoming the gold standard in combating ageism (WHO, 2021). In addition to the importance of research in campaign development and evaluation, community engagement with multiple stakeholders was identified as an essential component. Strategic planning and the implementation of key activities also were identified as important components that ensure the success of anti-ageism campaigns. Relying on adequate communication strategy, while communicating clear, evidence-based, and concise message also were found important (WHO, 2020).

In response to the WHO call to evaluate campaigns that tackle ageism (WHO, 2020), a recent study evaluated three different campaigns that took place in Israel during the COVID-19 pandemic. The authors found that all three campaigns focused on active, successful aging, thus presenting an overly positive biased perspective on older persons and older age as a continuation of middle age. Although the campaigns took place at the same period, they failed to combine efforts and each operated *in silo*, despite the common message conveyed by all three (Okun & Ayalon, 2022). A different study conducted in Lisbon, Portugal, described a public campaign to raise awareness of ageism and older age via billboards and posters. To assess the effects of the campaign, the authors interviewed varied relevant stakeholders which were all positive about the campaign (Mendonça et al., 2016). In addition to these documented efforts, multiple campaigns have taken place in the past decade. These campaigns were carried out by different non-governmental organizations committed to the topic of aging (e.g., the Centre for Ageing Better, AGE-Platform Europe, AARP).

### The present study

This study is a direct response to the need to raise awareness of the topic of ageism as a first step to tackling it. A recent survey of 1,025 Israeli respondents has found that 45% of them were not familiar with the term ageism. These individuals also were significantly less likely to acknowledge the experience of ageism. However, after receiving information about the term ageism, these individuals were more likely to acknowledge the experiences of ageism. Hence, this study highlights the need to ensure that people are familiar with the term (Okun & Ayalon, 2024).

Capitalizing on existing evidence on the efficacy of educational interventions in reducing ageism (Burnes et al., 2019; Lytle & Levy, 2019), the researchers developed a website called, “A site for all ages” (no2ageism.com). The overarching goal of the website is to provide evidence-based information about ageism in Hebrew and in English. This study reports on a strategy used to increase traffic to and engagement with the website. In Study 1, the researchers relied on a real-life experiment in which people were exposed to one of two ads to attract traffic to the website. The researchers did not embark on a campaign with a direct ad which explicitly invited people to learn more about ageism given our preliminary survey, which has shown that a large portion of the population is unaware of the term (Okun & Ayalon, 2024). Instead, the ads chosen for the campaign aimed to raise awareness of age discrimination in different ways: One of the ads was positive, fostering a sense of inclusivity regardless of one’s age and the other ad was negative, excluding older persons because of their age. Although the framing effect of positive versus negative messages on decision making has been studied for over four decades, exact predictions are still equivocal (Gong et al., 2013; Tang, & Chooi, 2023). Thus, the researchers had no hypotheses concerning the differential effect of the two ads. Whereas Study 1 was a real-life “natural” experiment, Studies 2 and 3 required participants to actively reflect on their choice of ads. In Study 2, the researchers queried those who clicked on the ad and entered the website about their decision to enter the website. Study 3 had the exact same purpose as Study 2 and was initiated due to the limited response to Study 2. In Study 3, the researchers relied on a survey company, which mailed an online survey, hypothetically asking respondents which of the two ads would be most appealing to them and why. Whereas Study 1 provides real-life input on the effectiveness of the ad campaigns, Study 2 provides a more in-depth exploration of people’s motives or rationale for entering the website. As response rate to Study 2 was limited (only 26 people out of 1,742 individuals who clicked on the ad), Study 3 was initiated to provide in-depth explanations from a larger number of participants. It also enhances the validity of the findings as it was conducted with a different sample, obtained via a paid survey agency, rather than a paid ad campaign, yet asked a similar question to Study 2. The researchers relied on a mixed-methods design, including quantitative data concerning the effectiveness of the ads to attract people to the website and people’s engagement with the website (Study 1), supplemented by more in-depth qualitative analysis of people’s reflections concerning the ads (Studies 2 and 3).

This study is important for the systematic approach it brings into campaign evaluation and for the practical insights gained by employing such a detailed approach. Following the WHO recommendations for the successful execution of social campaigns (WHO, 2020), the present campaign was supported by a research component from its early stages of initiation, the researchers fostered community engagement through all stages of the campaign, developed and tested a strategic plan for engagement, implemented key activities to mobilize engagement, and relied on a detailed communication plan as described in the Methods section. In the absence of past research on the topic, the researchers had no concrete hypotheses, but rather posed the following questions: (a) Can increased traffic also result in increased engagement with the website? (b) Is one ad (positive vs negative message) more likely to attract traffic than another? (c) Are people of certain characteristics more likely to be attracted to one ad over the other?

## Method

**Advisory team.** Prior to embarking on the development of the website and the campaign, the researchers established a team of social activists in the field of aging. The team consisted of eight individuals from the following fields: A CEO of an NGO in the field of aging, a city planner who specializes in old age, the Director of the Senior Citizens Department at the Ministry for Social Equality, a marketing specialist, two media specialists, and the two authors of this article, who are researchers with expertise in communication, psychology, and gerontology. Two of the team members self-identified as older persons over the age of 70 years. The team was established in response to the COVID-19 pandemic which brought with it a surge in ageism (Ayalon, 2020; Ayalon, Chasteen, et al., 2021). As part of the discussions, the team generated ideas to attract people to the topic of ageism using various ads. These ideas formed the basis for the two ads used to campaign for the website. The team also provided feedback on the website. The team convened 12 times between June 2020, and June 2021.

**Preliminary survey.** As part of their work with the advisory team, the researchers developed a research survey, which contained open-and closed-ended items to better understand the extent of knowledge and impact of ageism on lay people in Israel. The survey was administered to over 1,000 Israelis. Based on these data, the researchers published several articles concerning the preferred term to refer to older persons (Okun & Ayalon, 2023b), familiarity with the concept of ageism (Okun & Ayalon, 2024), and the importance of addressing self-ageism as a major hurdle in the lives of older persons (Okun & Ayalon, 2023a). These studies have inspired this study and strengthened our understanding that a national campaign to increase awareness of the topic of ageism is highly needed.

**Website design.** The website was developed in consultation with a web design company (Reut Tucker Studio) and the two researchers. In addition to the advisory team, several researchers in the field of social sciences (e.g., education, social work, music therapy, law) and experts in usability provided feedback on the website. The website covers topics such as the definition of ageism, its manifestations, ways to reduce it, and recent evidence on the topic. The website is available in Hebrew and English with a single introductory page in Arabic. For more information about the website, see no2ageism.com.

**Digital campaign.** The digital campaign consisted of a collaboration between the two researchers, a web design company, and a digital marketing expert (Lemesh Studio). The researchers utilized Google Ads, a paid advertising platform, to target their audience effectively. Google Ads allowed us to reach specific audience based on searched keywords in the field of aging (e.g., retirement, age, discrimination, older age, stigma), ensuring that their message was delivered to those most likely to engage with it. The campaign was conducted in Hebrew, with one of the two ads appearing to viewers, encouraging them to click and visit the website.

**Figure 1.**
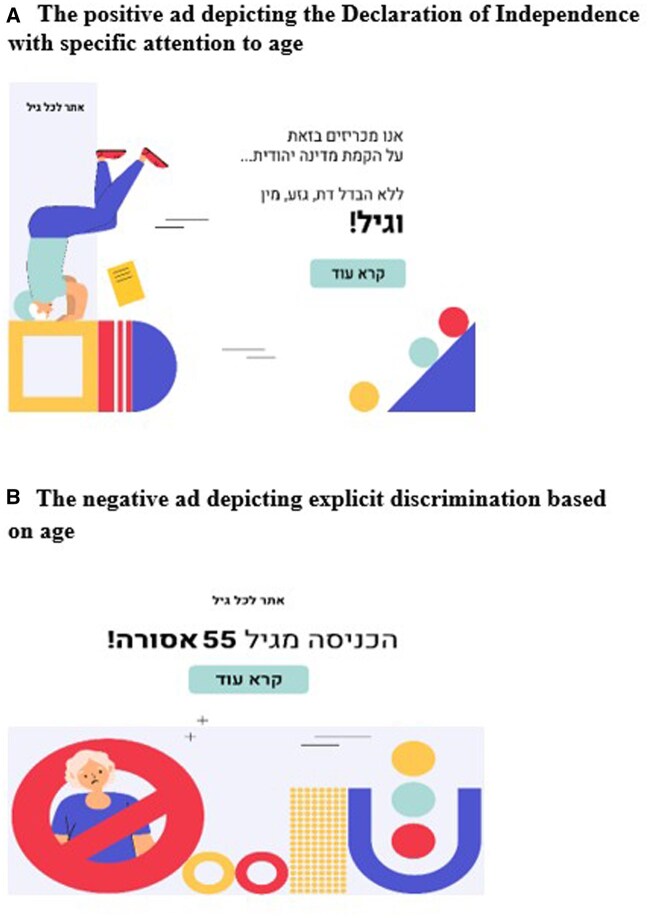
The positive and negative ads used to attract attention to the topic of ageism.

**Positive ad.** The ad features the well-known Declaration of Independence of the State of Israel, which opens with the following sentence: “We hereby declare the establishment of a Jewish state. No difference in religion, race, gender and… Age.” The addition of the word “age” to the values of inclusion required in a civilized country is a new idea, introduced by the ad. The illustration which accompanied the statement shows an older person standing on his head. This refers to the first prime minister of the State of Israel (David Ben Gurion), whose familiar nickname was “The Old Man,” an extraordinary leader who even in old age would make headstands on the beach. The overall message is that of inspiration but also nostalgia for an innocent period of hope with the expectation that people would be attracted to this message and click on the ad (see Figure 1a).

**Negative ad.** This ad introduced the text: “No entrance to people over the age of 55.” The visual image which accompanies the text shows a red traffic light which says, “no entrance” and an image of an older woman. The overall message is that of exclusion with the expectation that people would be curious enough to find out what is behind the exclusionary message (see Figure 1b).

**Landing page**. Each ad included a “Read more” button, so that by clicking on the ad, the viewer was led to the landing page of the campaign. The landing page consisted of three parts:

Part 1—A brief explanation of “what ageism is”: The idea was to explain to people that what they have just experienced through the ad are different forms of the inferno of ageism.

Part 2—An invitation to share ageist events and experiences they have encountered in life by clicking on a link and referring to the questionnaire.

Part 3—An invitation to visit the “Site for all ages” to continue browsing and receive additional information.

**Figure 2.**
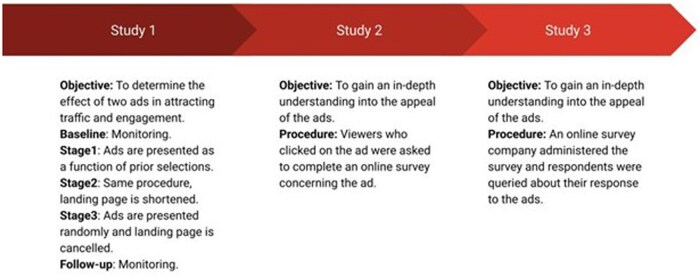
Study flow.

Figure 2 describes the flow of the three studies detailed in the next section and their main objective.

## Study 1

**Procedure:** The study received ethics approval from the PI’s university (#052303). Participants did not sign informed consent as no active data collection was conducted, other than the general privacy notifications employed by Google. To determine the effects of the two ads in attracting traffic to and engagement with the website, the researchers employed a trial which consisted of the following phases:

**Baseline:** The researchers collected baseline data on traffic and time spent on the website (e.g., session duration as an indicator of engagement) between August 15 and December 20, 2023.

**Stage 1:** The researchers determined the number of times each ad appeared as a function of prior selections of past viewers, so that an ad that received more clicks would be shown more. This stage took place between January 11 and February 5, 2024. Those who clicked on the ad landed on the website’s landing page.

**Stage 2:** The same procedure as in Stage 1, but the landing page was shortened to include only information about ageism and an invitation to review the website. The rationale for shortening the landing page stemmed from the limited engagement with the original landing page and attempts to attract visitors to further explore the website. This stage took place between March 6 and April 8, 2024.

**Stage 3:** The researchers randomly varied the presentation of the ads, but this time viewers had an equal chance to view one of the two ads, regardless of the number of previous clicks it had generated. The landing page was cancelled (given people’s reluctance to engage with the website), and people were directed immediately to the website. This stage occurred between April 14, and May 9, 2023.

**Analysis:** The researchers obtained descriptive statistics on the number of new users and average engagement time with the website at baseline, during the three stages of the experiment and at follow-up. For the three stages of the experiment, the researchers obtained information on Click Through Rate (CTR), which is the percentage of users who click on an ad in relation to the total number of ad views. The researchers also obtained descriptive information on age and gender by ad.

## Findings

There are several notable insights from Table 1. Overall, the CTR is around 1, with the lowest being for the negative ad in Stage 2, and the highest being for the positive ad in Stage 2. It appears as if in Stage 1, there was a preference toward viewing the negative ad, whereas in Stage 2, there was a preference toward viewing the positive ad. This resulted in Google Ads showing more of the negative ad in Stage 1 and more of the positive ad in Stage 2 (possibly supporting past inconclusive findings concerning framing effects; see Gong et al., 2013; Tang & Chooi, 2023). In Stage 3, the researchers ensured that both ads have an equal probability of appearing. This resulted in a slightly lower CTR overall. Although in Stage 1, it is the negative ad which has a higher CTR, in Stages 2 and 3, it appears that the positive ad has a higher CTR. With one exception (Stage 2, negative ad), the distribution of men versus women was almost equal in all three stages, regardless of the ad. Those 65+ were the ones most likely to click on the ad (regardless of its message), across all three stages of the experiment.

**Table 1.** Descriptive characteristics of website viewers exposed to the positive and the negative ads.

	Positive ad						Negative ad					
Experitmental stages	Number of views	Number of clicks	%CTR^a^	%Women^b^ of views	Age distribution of views (%)		Number of views	Number of clicks	%CTR	%Women of views	Age distribution of views (%)	
**Stage 1**	42,828	401	0.93	48	18–24	7.8	137,992	1,341	0.97	50	18–24	9.5
					25–34	11.6					25–34	12.2
					35–44	19.3					35–44	20.0
					45–54	14.3					45–54	14.7
					55–64	10.4					55–64	10.5
					+65	27.8					+65	25.3
					UA	8.6					UA	2.6
**Stage 2**	228,132	2,471	1.08	49	18–24	8.7	51,717	304	0.59	43	18–24	6.9
					25–34	15.8					25–34	12.2
					35–44	18.9					35–44	18.1
					45–54	12.3					45–54	13.2
					55–64	8.5					55–64	8.3
					+65	23.3					+65	31.9
					UA	4.8					UA	3.9
**Stage 3**	126,497	1098	0.87	48	18–24	8.9	113,175	911	0.80	50	18–24	8.8
					25–34	13.6					25–34	13.5
					35–44	18.6					35–44	19.1
					45–54	13.4					45–54	14.0
					55–64	8.8					55–64	8.5
					+65	28.6					+65	28.6
					UA	7.9					UA	7.4

UA = unavailable.

a Click through rate.

b Numbers may not sum as age was unavailable for some participants (for both positive and negative ads).

As presented in Table 2, *at baseline*, the average number of visits was 2.9 per day and *at follow-up*, the average number of visits was 4.6, indicating an increase in the number of visits. The average session duration *at baseline* and *at follow-up* was a little over two minutes. As to the experiment, *at Stage 1*, the average number of visits was 3.7 per day, *at Stage 2*, the average number of visits was 8.9 per day, and *at Stage 3*, the average number of visits was 47.9 visits per day (as there was no landing page at that stage). Engagement across the three stages was quite comparable and low, well-below one minute, attesting to the limited efficacy of the campaign in engaging viewers.

**Table 2.** Descriptive data on the number of new visits and average time spent on the website.

	New visits	Average visits per day	Average session duration (seconds)
**Baseline (August 15–December 20, 2023; 127 days)**	370	2.9	140
**Stage 1 (January 11–February 5, 2024; 25 days)**	130	3.7	21
**Stage 2 (March 6-–pril 8, 2024; 33 days)**	294	8.9	10
**Stage 3 (April 4–May 9, 2024; 35 days)**	1675	47.9	16
**Follow-up (June 1–September 30, 2024; 121 days)**	553	4.6	144

Overall, even though the paid ads were successful in generating traffic to the website, the researchers did not reach a conclusive decision as to which ad attracts more visits. Moreover, the researchers were unable to improve the engagement with the website, thus leading them to conclude that their efforts to produce an online campaign via the educational website were quite futile.

## Study 2

**Procedure:** This study was conducted in conjunction with Study 1. It received the ethics approval of the PI’s university (#052303). Participants received information about the study and its purpose and provided informed consent prior to participating in the survey. Viewers who clicked on the ad and landed on the landing page received information on the topic of ageism. Subsequently, they were asked to respond to the following questions:

1. Demographic information: age, gender, education
2. How did you get to this webpage? via the positive ad, negative ad, or random scrolling
3. What do you think of the ad? _____________

**Analysis:** We conducted basic descriptive analysis to better understand the characteristics of respondents and their experiences of ageism. Thematically, we reviewed the responses and attempted to identify a common thread.

## Findings

A total of 26 individuals responded to the survey (out of 1,742 individuals who clicked on the ad in Study 1), comprising 12 women, 5 men, and 9 who did not report their gender. The age range of respondents was between 16 and 74 years. Marital status varied, with four married, four widowed, four single, two divorced, and the remainder not reporting. Among the respondents, 19 clicked on the positive advertisement, while 7 clicked on the negative one. When asked why they clicked on the ad, responses were highly individualized. For instance, one respondent wrote, “I wondered if it was a progressive ad or just meant to grab attention,” while another stated, “I was curious,” or “I felt a connection.” Others mentioned feelings of confusion, such as “Strange…” or “I didn’t understand the message.” As the number of responses was low and variability was substantial, the researchers did not categorize the responses into thematic categories, though point that they mainly acknowledged curiosity as a reason for clicking the ad and entering the website.

## Study 3

**Procedure:** The study received the ethics approval of the PI’s university (#012409). An online survey company administered the survey between March 31  and April 14, 2024. Participants received information about the study and its purpose and provided informed consent prior to participating in the survey. Respondents received payment for their participation in the study.

**Sample:** The sample consisted of 908 respondents. Women constituted the majority of the sample (55.7%) and the average age of the sample was 47 (SD = 13.8). Most of the sample had an academic degree (50.3%).

**Survey:** Respondents were introduced to the two ads and were asked to choose the one they found most appealing. They were subsequently asked to explain their choice.

**Analysis:** The researchers used descriptive statistics to identify the characteristics of respondents who chose each of the ads. They utilized the “word cloud” interface to identify the most common words used in response to each of the ads. This approach provided a quick and straightforward way to visually summarize key themes, making it easier to capture the audience’s interest and enhance the communication of our research findings. Word clouds are particularly valuable for their ability to highlight recurring patterns within qualitative data, serving as an initial step before a more detailed analysis takes place (Heimerl et al., 2014). In addition, the researchers conducted a thematic analysis (Robinson, 2022) to further explore the main themes generated in response to the ads. They began by generating five to six codes based on similar types of answers, with the codes representing potential themes (e.g., curiosity, anger, mistake, better than the other ad, identify with the message). Codes that did not appear frequently across the responses were discarded, while those that occurred multiple times were consolidated into distinct themes. These recurring themes then became key findings in the research, providing deeper insights into the reasons behind participants’ ad preferences.

The analysis was facilitated by Atlas.ti, a software program designed for qualitative data analysis. Atlas.ti enables researchers to systematically analyze large volumes of text, images, audio, and video data by organizing and categorizing it into codes and themes. The program’s tools for coding, visualizing, and querying data made it easier for us to identify patterns, relationships, and key insights within the participants’ responses. The reliance on two independent researchers, who analyzed the data, and two different research methods to analyze the responses provides a means of triangulation, which enhances the trustworthiness of the findings. As the texts provided by participants were very short and succinct, the coding resulted in almost perfect agreement between researchers once the exact coding system was established. Disagreements were resolved in a discussion, which also resulted in the decision to present only the most frequent codes.

## Findings

Most of the sample (*n* = 736; 81%) chose the positive ad over the negative one (*n* = 172, 18.9%). Those who chose the positive ad were significantly older (*M* = 48.8, *SD* = 18.1) than those who chose the negative one (*M* = 40.0, *SD* = 17.6; *t* [905] = 5.70, *p* <.001). Women were significantly more likely to choose the positive ad compared with the negative ad (58% vs 42.4%; Chi^2^[1]=15.18, *p* <.001). Those who chose the positive ad had a higher level of education compared with those who chose the negative one (elementary school: .5% vs 2.9%; academic degree: 52% vs 43%, respectively; Chi^2^[3] = 11.63, *p* <.01).

**Figure 3.**
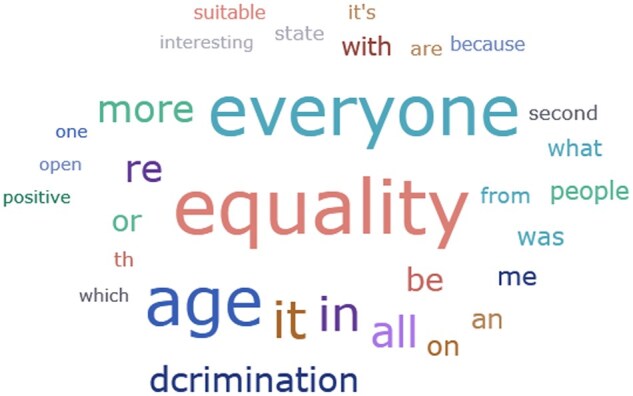
Word cloud in response to the positive ad.

**Reasons for choosing the positive ad:** The top three words to explain their choice among those who chose the positive ad were: All/everyone, age, and equality (see Figure 3).

An in-depth analysis of the responses revealed that the top three reasons given for choosing the positive ad were because it:

1. Elicits a sense of inclusiveness and togetherness (*n* = 460): “Equality, a mutual fate;” “Openness and inclusion;” “Everyone is equal;” “Fits all ages, all human beings.”
2. Is colorful and funny (*n* = 122): “Very nice;” “Sweet;”” Happiness to all;” “Beautiful colors, interesting;” “Colorful, interesting graphics.”
3. Elicits curiosity (*n* = 42): “Curiosity;” “More interesting- to understand what they (advertisers) are implying;” “Wanted to know what’s behind. What is the ad for;” “Made me curious;” Interesting;” “An interesting and innovative concept.”

**Figure 4.**
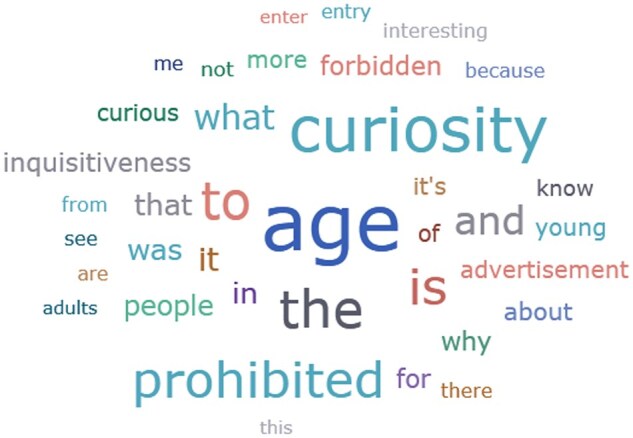
Word cloud in response to the negative ad.

**Reasons for choosing the negative ad:** The top three words to explain their choice among those who chose the negative ad were: Curiosity, age, and prohibited (see Figure 4).

An in-depth analysis of the responses revealed that the top three reasons given for choosing the negative ad were because it elicits a sense of

- Curiosity and interest (*n* = 96): “A wish to see what is prohibited (people over 55);” “Curiosity;” “I was curious to know what’s the content of the website;” “It has the opposite effect-making people curious and wish to know what’s prohibited;” “Curiosity-testing limits.”
- Anger and repel (*n* = 36): “Because it (ad) elicits antagonism;” “Repelling;” “Illegal;” “Someone is trying to annoy on purpose;” Anger—I would enter to see what’s behind this nonsense- no entry over a certain age.”
- No real reason for choosing one over the other (*n* = 22): “No real reason;” “Nothing;” “The truth is that I wouldn’t enter either one, but I had no choice;” “No concrete reason.”

## Discussion

Ageism poses a major public health threat with one in two people reporting being ageist (Officer et al., 2020) and one in three reporting being exposed to ageism (Ayalon, 2014). Ageism has a dramatic impact on the health and wellbeing of older persons as well as on the economy (Chang et al., 2020; Levy et al., 2020). Despite the WHO’s efforts to advocate for intervention research, arguing that “the top-most priority should be developing strategies to reduce ageism” (WHO, 2021, p. XVII), which build on existing evidence and scale up to impact the entire population, research concerning the use of campaigns to tackle ageism is still lacking (WHO, 2020). Hence, this study is a harbinger of systematic research to develop evidence-based social campaigns to tackle ageism. Procedurally, the study follows guidelines produced by WHO for the development of social campaigns to tackle ageism (WHO, 2020). Specifically, we collaborated with a large and diverse number of stakeholders in the production of the campaign and the website, ensured that the campaign was informed and accompanied by research, planned the campaign strategically, and attempted to produce effective communication messages.

**Can increased traffic also result in increased engagement with the website?** Based on Study 1, the CTR across the three phases roughly varied around 1. This is comparable to past research which has shown variability across CTR depending on the industry, with real estate ads generating a CTR a little over 1 and other industries generating a CTR lower than 1 (CXL, 2024). Hence, it is safe to conclude that the campaign was effective in increasing traffic to the website. Moreover, a comparison between baseline (no paid ads) and follow-up (paid ads) shows an increase in the number of new users, suggesting that overall interest in the topic has increased over time. Although many other explanations, such as ongoing educational interventions to address ageism at the local level (Suberry et al., 2025) or the WHO global campaign (WHO, 2021), could have accounted for the increased traffic to the website, the ad campaign could possibly be one such explanation.

Given the fact that a few hundred thousand people viewed the Google Ads, resulting in an acceptable CTR, it can be concluded that the ads were well-designed and effective in attracting viewers. However, this proved to be insufficient. The campaign fell short in that only a small portion of the public who was exposed to the ads remained on the website to read the landing page, very few completed the survey, and most visitors either left the website quickly or did not engage for a meaningful duration. Hence, the main goal to increase interest in and familiarity with the concept of ageism via exposure to the website has failed in the face of only very limited time spent on the website by random viewers. This is consistent with past research which has argued that clicking on an ad does not necessarily translate into purchasing a product (Dalessandro et al., 2015). As the campaign took place after October 7, being in a state of constant terror and war could at least partially explain the failure to engage the public (Yavetz et al., 2025). It also is possible that the website was not engaging enough despite attempts to consult with professionals and the reliance on feedback from multiple sources to inform its creation.

The nature of ageism may also explain the somewhat limited interest in the website found in this study. Even though ageism is more prevalent than sexism or racism (Ayalon, 2014), there is substantially more research on the latter two “isms” (Nelson, 2005). Hence, even the scientific research community is less tuned in to understanding the topic of ageism. Possibly behind the limited awareness of the term, found in past research (Okun & Ayalon, 2024), there might be a bigger barrier than simply lack of information. Ageism may not be as appealing as sexism or racism because it represents our fears of our own aging (Nelson, 2005). This is in contrast with sexism or racism which are usually directed externally toward “the other” and therefore can be seen as less threatening.

**Is one ad (positive vs negative message) more likely to attract traffic than another?** Based on Study 3, those who clicked on the positive ad attributed this to the fact that the ad gave them a sense of inclusiveness and equality. Some also acknowledged the fact that the ad addressed age issues as an appealing factor. In contrast, the main reasons for choosing the negative ad were curiosity, followed by anger and repel for introducing such a negative message. Hence, the main reasons provided by respondents were in line with our initial design approach, suggesting that the viewers were able to grasp the intended message behind each of the ads.

Relying on survey data, the statistical difference obtained in favor of the positive ad, coupled by the qualitative difference in responses to the two ads clearly show that respondents were able to distinguish the positive from the negative. However, in real life (Studies 1 and 2), these differences were somewhat less clear-cut, as no definite conclusions can be drawn about individuals’ preferences. In Stage 1 of Study 1, there was a preference toward the negative ad, resulting in Google’s optimization showing the negative ad more frequently, but in Stage 2 of Study 1, the preference was toward the positive ad. However, with one exception, the CTR was higher for the positive ad suggesting that it is more likely to generate people’s engagement.

The findings possibly point to the undesired nature of ageism (Ayalon & Tesch-Römer, 2018). When brought into one’s conscious awareness with a concrete request to make an explicit choice between two options, people were able to make the “correct” choice and to favor the optimistic, egalitarian ad. However, when faced with a single choice under the privacy of the internet, “curiosity takes over” at times, and people may choose ads that they easily identify as negative or harmful.

**Are people of certain characteristics more likely to be attracted to one ad over the other?** Across all stages of the experiment of Study 1, the older age group of people over the age of 65 years was most likely to click on the ad and view the website. This is contrasted with the 18–24 age group, which was the least likely to click on the ad and view the website, regardless of the message featured by the ad. These findings are consistent with past research which has shown that in general, older persons are more likely to respond to online communications (Marketing Charts, 2019) and even to uncritically accept online fake news (Ayalon, 2024). As the messages communicated by the ads might be particularly relevant to older people, this might be another incentive for them to click the ad.

Based on the survey data (Study 3), respondents were significantly more likely to report a preference toward the positive ad. Specifically, women, older persons, and more educated individuals were more likely to report a preference toward the positive ad which instigated a message of inclusion and equality. As women and older persons are more likely to be exposed to ageism (Handy & Davy, 2007; Krekula et al., 2018), this is not a coincidence. In addition, more educated individuals might be more tuned toward the identification of negative ageist messages than less educated individuals. This has been shown in past research, with more educated people being less likely to report ageist sentiments, compared with less educated individuals (Allen et al., 2023).

**Limitations and implications for research and practice**. Although both the website and the ads were developed through a collaborative, iterative process, there is always the chance that better designs, better messages, or more money put into the campaign would have resulted in more positive outcomes. The two ads that were examined in this study varied not only by content, but also by visual design and implicit messages incorporated in them. Hence, further research might benefit from a more careful examination of positive versus negative messages by varying a single domain at a time. It also is important to stress once again the fact that the study was conducted during unordinary times in a country that is fighting for its existence (Greenland et al., 2024). Issues such as equality, and inclusiveness have major implications on the one hand, especially given the protests in response to increasing inequality and violation of human rights by the government that have been taking place in the country for almost two years now (Ayalon & Okun, 2024), but on the other hand, might be seen as less important especially given ongoing threats to one’s physical existence and to the existence of Israel as a democratic State.

Despite its limitations, the study has several implications for further research and policy. First and foremost, we believe the efforts described in this article to develop and evaluate a national campaign to educate the public about the phenomenon of ageism is commendable. As already noted, the WHO had issued a call to the scientific community to develop a more comprehensive body of knowledge on interventions to reduce ageism, with a special focus on social campaigns, which are currently lacking evidence (WHO, 2020). Even failed attempts are important to report because a) they can deter researchers and practitioners from following unsuccessful pathways, and b) they may instigate researchers’ and practitioners’ curiosity and imagination to develop more successful means to educate people about ageism at the public level. Our findings highlight the challenges faced by researchers translating research findings into real life application. They call for more real-life research in the field to possibly implement and adopt lessons learned under lab conditions. Practically, it shows the long way to go from production to “selling” of a social product. It also alludes to the fact that there is a big difference between instigating an initial interest and engaging people in a topic. We believe that our detailed and elaborate methods should be replicated in future campaigns, which may result in more positive outcomes.

In reviewing the findings, it is possible to argue that ageism is a challenging, possibly less attractive topic to engage random people in. This can be contrasted with more successful campaigns previously reported in other fields (Eduzor, 2024). However, it is also important to recognize the tendency to publish and acknowledge successful campaigns and to disregard unsuccessful ones. Hence, it is highly likely that for every “success,” there are multiple failures or challenges not adequately monitored and discussed in the literature, possibly hindering our ability to advance.

## Data Availability

Data are available upon request and upon the approval of the Institutional Review Board.
